# Revisiting Mitochondria Scored Cancer Progression and Metastasis

**DOI:** 10.3390/cancers13030432

**Published:** 2021-01-23

**Authors:** Rohit Gundamaraju, Wenying Lu, Rishya Manikam

**Affiliations:** 1 ER Stress and Mucosal Immunology Lab, School of Health Sciences, College of Health and Medicine, University of Tasmania, Launceston, Tasmania, TAS 7248, Australia; 2 Respiratory Translational Research Group, Department of Laboratory Medicine, School of Health Sciences, University of Tasmania, Launceston, Tasmania, TAS 7248, Australia; wenying.lu@utas.edu.au; 3 Emergency and Acute Care Centre, Faculty of Medicine, University Malaya, Kuala Lumpur 59100, Malaysia

**Keywords:** mitochondria, metastasis, OXPHOS, cancer, Warburg effect, cancer therapeutics

## Abstract

**Simple Summary:**

The indispensible role of mitochondria has been described over a century ago by Otto Warburg which has been serving the fields of cell biology and cancer biology immensely. Mitochondria are the principal site for vital mechanisms which vastly dictate the physiology. The intricacy of mitochondria’s role cancer have been noticed and well addressed in recent times. The underlying mechanisms are surfacing to unveil the nature of mitochondria and its participation in tumor cell motility and metastasis. This addressing may unravel novel therapeutic options. This review summarizes and reweighs the key aspects like underlying and emerging mechanisms which might be useful in designing novel chemotherapy.

**Abstract:**

The Warburg effect has immensely succored the study of cancer biology, especially in highlighting the role of mitochondria in cancer stemness and their benefaction to the malignancy of oxidative and glycolytic cancer cells. Mitochondrial genetics have represented a focal point in cancer therapeutics due to the involvement of mitochondria in programmed cell death. The mitochondrion has been well established as a switch in cell death decisions. The mitochondrion’s instrumental role in central bioenergetics, calcium homeostasis, and translational regulation has earned it its fame in metastatic dissemination in cancer cells. Here, we revisit and review mechanisms through which mitochondria influence oncogenesis and metastasis by underscoring the oncogenic mitochondrion that is capable of transferring malignant capacities to recipient cells.

## 1. Good and Bad Mitochondria

**Figure 1:**
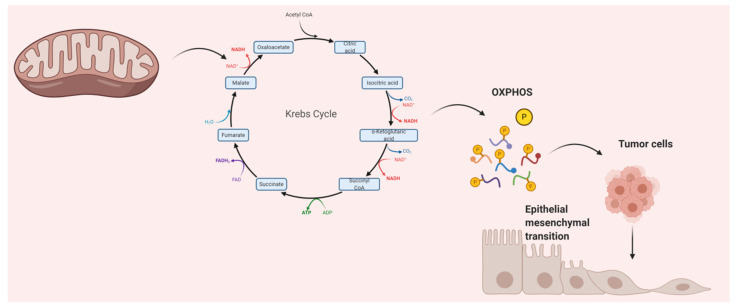
Epithelial–mesenchymal transition (EMT) by mitochondria.TCA: tricarboxylic acid; OXPHOS: oxidative phosphorylation. Mitochondrial metabolites are accumulated upon mutation of the indicated TCA cycle enzymes which activates the EMT. In cancer cells, the TCA cycle not only serve to produce reducing equivalents to fuel the electron transport chain, but also to generate biosynthetic intermediates that are necessary for cell proliferation and migration.

Tumor cell metabolic reprogramming dictates the difference between normal and tumor cells. Mitochondria play a major role in metabolic reprogramming:It has been shown that tumor mitochondria not only change their structure but also decrease the potential of oxidative phosphorylation (OXPHOS) and apoptosis [1]. Considering the countless functions of mitochondria, including in the tricarboxylic acid (TCA) cycle, OXPHOS, etc., it is no surprise that mitochondria are directly involved in cancer progression (Figure 1) [2]. Metastasis is a hallmark of cancer and includes several steps: detachment of local tumors, intra-invasion, circulation in the blood, extra-invasion, and colonization in the secondary sites for survival. In all the above stages, mitochondrial metabolism is tuned for tumor cell adaptation to facilitate metastasis [3]. In addition, several postulations have been proposed on the vital role of mitochondria in metastasis, where mitochondria help overcome perturbations in metastasis environments. mtDNA single nucleotide polymorphisms (SNPs) and a few mutations might lead to distinctions in metastatic susceptibilities in cancer histotypes or patient groups. Many studies have revealed that mitochondria are involved in a chain of events including modulation of the the microenvironment, motility and invasion, plasticity, and consolidation [4]. Therefore, in the current review, we consolidated the essential contributing factors of mitochondria in cancer progression and, specifically, metastasis. We also discuss several questions that address the underlying mechanisms of context-dependent contributions of mitochondria in metastasis. 

## 2. Can Mitochondrial Dynamics Dictate Cancer Spread?

**Figure 2:**
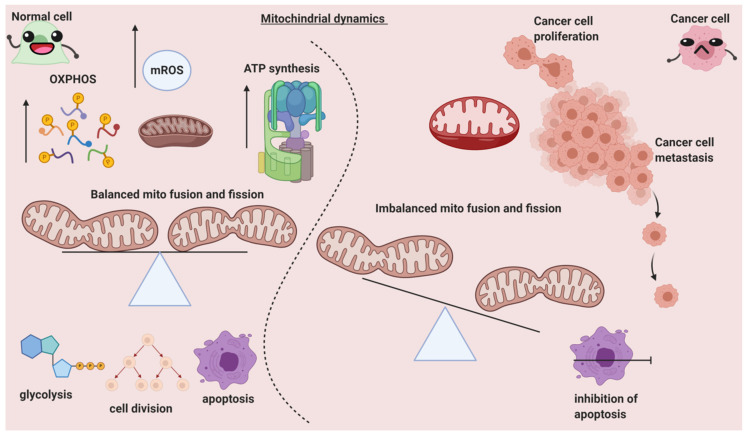
The differential role of mitochondrial dynamics in normal and cancer cells.OXPHOS: oxidative phosphorylation; ATP: adenosine tri phosphate; mROS: mitochondrial reactive oxygen species. The morphology and physiology of the mitochondria and its healthy functioning is governed by fission proteins. Mitochondria fission promotes glycolysis, mitophagy, and apoptosis and is also necessary for cell division. In contrast, mitochondria fusion promotes ATP and ROS production via OXPHOS. In normal cells (left area of the figure), mitochondria fusion and fission are well-balanced, which results in healthy homeostasis. Whereas in cancer cells, an imbalance of the fusion and fission is favored to drive proliferation, metastasis, and the maintenance of cancer stem cell phenotypes.

The balance of mitochondrial fusion and fission is necessary for the regulation of various processes, including the quality of mitochondria, cell metabolism, cell death, proliferation, and cell migration, and is maintained by numerous mitochondrial-shaping proteins. Negative modulations or malfunctions in these processes resulting in changes in mitochondrial dynamics lead to diseases like cancer [5]. Mitochondrial dynamics are correlated with various diverse disease pathologies. For instance, a high nutrient-deprived state triggers mitochondrial fission and hence results in programmed cell death. A significant number of proteins such as the GTPases (Mfn1, Mfn2, Opa1, and Drp1) have strong regulatory effects in balancing mitochondrial fusion and fission. Any perturbations or failure in managing the correct dynamic state leads to cancer [6]. It is noteworthy that mitochondrial dynamics have a deep role in cancer cell migration (Figure 2). The discovery of susceptibility to cancer associated with altered or modulated mitochondrial dynamics could result in new targeted therapies. Some of these altered dynamics, such as mitochondrial fission, are discussed in this review.

Mitochondrial dynamics also massively impact apoptosis. Studies have correlated them to dynamic homeostasis and tumor growth. Strikingly, signaling downstream of mutant KRAS in pancreatic cancers leads to mitochondrial fragmentation and increased activation of Drp1, processes that are required for KRAS-driven tumor growth in vivo. In addition, recent studies also suggest that mitochondrial dynamics are important for regulating metastatic phenotypes such as invasion and migration in breast and thyroid cancers [7].

Examples of high-to-low expressions of Drp1 and Mfn1 have been implicated in metastatic breast cancer [8]. Moreover, Drp1 is regarded as crucial for apoptosis due to its informed role in releasing cytochrome-c. The Drp1/Mfn1 expression ratio correlates to aggressive cancers and cell proliferation. The Drp1/Mfn1 expression ratio was found to be increased in hepatocellular carcinoma (HCC) tissues and associated with poor prognosis. Escalated mitochondrial fission mediated by imbalanced reactive oxygen species (ROS) production was found to be the primary reason for the pro-survival ability of the HCC cells under both in vitro and in vivo conditions [9]. High Drp1 expression was also observed in ovarian cancers where Drp1 was found coexisting with cell cycle-related genes, thereby facilitating cancer cell proliferation [10]. Mitochondrial fission also aided in cisplatin resistance in ovarian cancers [11]. Drp1 inhibitors like Driptor1 were employed against breast cancer cells, which show not only that the mitochondrial dynamics-mediated pathway is useful in designing anti-cancer therapy [12] but also that mitochondrial fission facilitates the survival, apoptosis, and drug resistance of breast cancer cells [13].

Mammalian target of rapamycin complex 1 (mTORC1), which is a trigger factor in cancers, stimulates translation of mitochondrial fission process 1 (MTFP1), which is coupled to pro-fission phosphorylation and mitochondrial recruitment of DRP1 in melanoma cells [14]. This shows that DRP1 couples with pro-cancer pathways. Other very recent evidence of mitochondrial dynamics and their role in cancer promotion was recorded in a study where Drp1 increased prostate cancer cell survival under metabolic stress conditions [15]. Further, knock down of Nestin, which is one of the classic markers in gastrointestinal cancers, downregulated recruitment of Drp1 to mitochondria in gastrointestinal stromal tumor cells [16]. To support this, another novel study consisting of Paris Saponin II (PSII), a major steroidal saponin extracted from Rhizoma Paris polyphylla, was employed against Drp1, which aided the modulation of Drp1-mediated mitochondrial fission [17]. The PSII in [17] surprisingly downsized the xenograft tumor size and impeded the phosphorylation of ERK1/2 and Drp1 at Ser616. Mitochondrial dynamics also enormously influence survival and stemness maintenance of cancer stem cells (CSCs), which are responsible for tumor recurrence and other malignant traits. Blockade of fission debilitated the self-renewal capacity of CSCs and led to CSC exhaustion. In addition, the reliability and functionality of T cells in the cancer microenvironment depend vastly on the mitochondrial dynamics balance, which hints at the essentiality and usefulness of targeting mitochondrial dynamics in anti-cancer treatment [18].

## 3. Mitochondria’s Vital Role in Numerous Cancers

Mitochondria have been well-known as crucial factors in various characteristics of cancer biology, including cancer development, metastasis, and drug resistance [19,20]. The alteration of mitochondria dynamics can affect the regulation of cancer cells. Mitochondria duties in dynamic networks include changes in size and distribution of sub-cellular components, and these dynamics are maintained by two main opposing processes: fission and fusion [21], regulated by dynamin-related protein 1 (Drp1) and mitofusins (Mfns) [22], respectively. Unbalanced mitochondrial fission or fusion dysregulates the cellular processes that contribute to tumorigenesis [23,24]. In breast cancer, increased mitochondrial fragmentation intensifies the capabilities of breast cancer cells to metastasize by activating Drp1 or silencing Mfns [8].The imbalance of Drp1/Mfn expression has also been found to cause additional mitochondrial fission and impaired mitochondrial fusion inhuman lung cancer cell lines, which is a key process for cell cycle progression [25].In addition, cancer cells are involved in the mitochondrial respiration chain to gain an obvious increase in ATP production [26]. Cancer cells generate invasion or metastasis by utilizing energy, powered through the transcription co-activator, PGC-1α, to promote OXPHOS, mitochondrial biogenesis, and oxygen consumption rate [27]. The association between PGC-1α expression in the invasion and metastasis of human invasive breast cancer was found in previous study [27]. Furthermore, the dysregulation of mitochondrial respiratory chains prompts ROS-induced integrin β5 expression and results in an increase in tumor cell invasion and metastasis in gastric cancer cells [28]. In addition, mitochondrial respiratory chain complexes are involved in cell apoptosis processes, in particular, complexes I and III are key regulators of cell apoptosis and major sources of ROS generation [29]. In most aggressive breast cancer, the most remarkable activity of complexes is observed. ROS-associated signaling pathways can be a potential suppressor for the tumor treatment target. On the other hand, mitochondrial dysfunction is identified as being associated with cancer progression. mtDNA mutations have been frequently encountered in cancer cells. Mitochondrial fusion activity is essential for mtDNA maintenance, a loss of mtDNA has been correlated with the drug resistance of anti-estrogen therapy in breast cancer [30]. Moreover, the mutation of mtDNA is one of the key factors that stimulate mitochondrial-mediated metastasis. For instance, mutated ROS-generating mtDNA promotes invasion and metastasis in lung cancer cells and breast cancer cells [31,32]. In addition, declined OXPHOS gene expression was found to result in metastasis in cancer cell lines and in metastatic melanoma in renal cancer specimens [33]. 

Other aspects to be considered are cross-links with mitochondrial dysfunction and promotion of tumor cells metastasis. Epithelial–mesenchymal transition (EMT) enables cancer cells to obtain the migration abilities to move out of the primary tumor and translocate to new target organs [34,35]. EMT transfers the epithelial cell to mesenchymal phenotypes in many epithelial tumor cells that are affected by mitochondrial dysfunction [33]. Mitochondrial dysfunction initiates EMT via EMT signaling pathways. TGF-β is known as a key growth factor controlling EMT progression through TGF-β/SMAD/SNAIL, phosphatidylinositol-3-kinase (PI3K)/AKT signaling pathways [36]. TGF-β phosphorylates TGF-β receptor-regulated Smad2 and Smad3, then upregulates the expression of their downstream gene, Snail-1, which is a positive regulator of EMT and metastasis [36]. Activated PI3K/AKT signaling can also upregulate the intracellular expression of Snail, thereby inducing the EMT [37]. The depleted mtDNA induces mitochondrial dysfunction and further triggers EMT induction, the prostate and breast adenocarcinoma cells show mesenchymal phenotypes with TGF-β overexpression [38].Moreover, in hepatocellular carcinoma cells, mtDNA depletion induces EMT via TGF-β/SMAD/SNAIL signaling [39]. In the tumor microenvironment, the hypoxia-induced accumulation of HIF-1 alpha activates the expression of TWIST which ultimately induces EMT [40]. The co-expression of HIF-1 alpha, TWIST, and Snail in primary tumors of head and neck cancer patients correlates with the poorest prognosis [41]. mtDNA depletion also can induce mitochondrial dysfunction and promotes EMT induction via mitochondrial reversed signaling. Mitochondrial reversed signaling triggers transcriptional activation of EMT signaling pathways, such as SNAIL, TWIST, and mesenchymal markers, such as vimentin, N-cadherin, with a corresponding loss of epithelial marker E-cadherin [42]. mtDNA-depletioncan also cause a loss of mesenchymal phenotypes of ESPR, such as ESPR1 in breast cells and expressed stem-cell phenotypes, suggesting a generation of cancer stem cells [42]. On the other hand, mutated mitochondrial metabolic enzymes are closely correlated with EMT-induced metastasis, which contributes to the initiation of oncogenic signaling cascades in cancers [43,44]. Another link with mitochondria in cancer cell metastasis is epidermal growth factor receptor (EGFR). EGFR was found intensively expressed in the mitochondria of highly invasive non-small cell lung cancer (NSCLC) cells [45]. EGF is a growth factor that initiates the EMT by activating the RAS/RAF/MEK/ERK MAPK signaling cascade. The activated ERK1/2-MAPK induces EMT, promoting the regulation of cell motility and invasion [46]. EGF initiates cancer cell invasion by regulating mitochondrial functions. EGF activates the mitochondrial translocation of EGFR, mitochondrial fission, and redistribution, upregulates cellular ATP production, and enhances cancer cell motility in vitro and in vivo. Furthermore, EGFR can regulate mitochondrial dynamics by interchanging with Mfn1 and disturbing Mfn1 polymerization, therefore, overexpression of Mfn1 reverses the phenotypes resulting from EGFR mitochondrial translocation to induce mitochondrial fragmentation [45]. 

## 4. Multiple Mechanisms of Metastasis by Mitochondria

Deciphering the mechanisms of metastasis involving mitochondria is extremely important in establishing therapeutics. The tumor microenvironment plays a prominent role in the progression of cancer, and it has a similar role in cancer chemoresistance via a mechanism called mitochondrial transfer, which broadly favors further invasion and metastasis. Mitochondrial transfer occurs in cells that fail to perform aerobic respiration due to mtDNA malfunction [47]. On the other hand, a horizontal mitochondrial transfer is also associated with chemoresistance. In the tumor microenvironment, horizontal transfer is regarded as lethal, since the transfer of mtDNA from the host cell to the cancer cell leads to escalated tumor-initiation ability because the cancer cells possess reduced respiratory function, and horizontal transfer in such instances improves the aggressiveness of cancer cells. Studies have exhibited that mtDNA transfer protects the cells from chemotherapeutic drugs. In a study involving acute myelogenous leukemia (AML), cells took up functional mitochondria from the bone marrow-derived stromal cells, which lead to protection of the cells from the drug effect and evasion of cell death [48]. Mitochondrial transfer under an in vivo setting not only leads to chemoresistance but also disease relapse. This entire concept of mitochondrial transfer endorses the notion of tumor plasticity and highlights the ability of the tumor cells to overcome unfavorable conditions by altering energy metabolism [19]. Further, mitochondrial transfer has been implicated in murine tumor models with essential functional consequences for tumor growth and metastasis. This has also been supported by studies where the mitochondrial transfer rescued cancer cells that were suffering deficiencies in OXPHOS and were prone to therapeutic apoptosis [49]. It is a proven phenomenon that dysregulated mitochondrial trafficking leads to metastasis of cancer cells.

**Figure 3:**
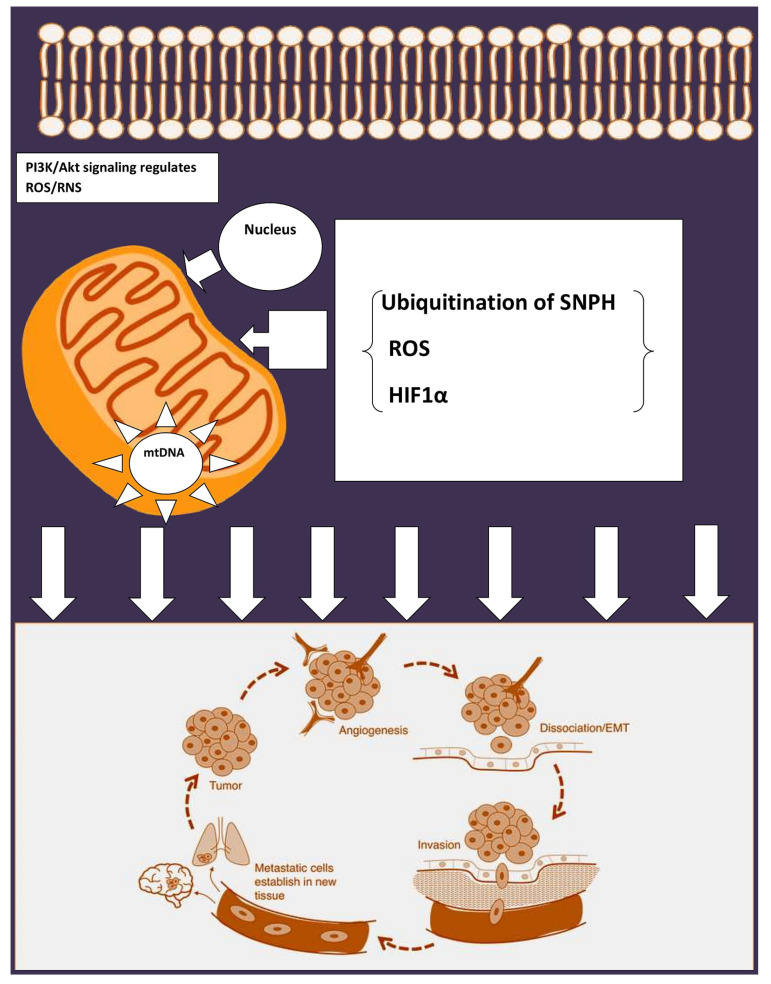
Regulation of EMT by mitochondria.ROS: reactive oxygen species; PI3K: Phosphoinositide 3-kinases; SNPH: syntaphilin; HIF: hypoxia inducible factor. Tumorigenesis calls for hypoxic-mediated reprogramming for metastasis. Bcl-2 family members regulate the PI3K pathway involved in metastasis progression. ROS generated during the metabolic process play a critical role in metastasis. Syntaphilin (SNPH), which generally arrests the mitochondrial trafficking in neurons, inhibits metastasis. In tumors with high expression of SNPH, mitochondria are anchored perinuclearly, resulting in lessened cell invasion and inhibited metastatic dissemination. In tumors with loss of SNPH expression, mitochondria are free to move to the cortical cytoskeleton via Kinesin/MIRO1 complexes. These cortical mitochondria fuel enhanced tumor cell invasion and correlate with poorer prognoses. Hypoxia can increase eNOS phosphorylation by activating the PI3K/AKT pathway. HIF-1α can also directly influence the expression of eNOS, which can be activated by phosphorylation of the serine 1177 residue, thereby, triggering migration and angiogenesis.

Ubiquitination of syntaphylin (SNPH) (Figure 3) is regarded as a vital regulator of mitochondrial trafficking. Studies show that SNPH aids in binding the mitochondria to the microtubule. Mechanistic studies hint that SNPH is modified by the ubiquitin ligase CHIP/STUB1 and deubiquitinated in a USP7-dependant manner, which suggeststhat ubiquitination of SNPH isa pivotal regulator of mitochondrial trafficking and tumor cell invasion [50]. Apart from the SNPH mechanics, hypoxia also governs mitochondria localization in cancer cells. Tumor cells under the influence of hypoxia downregulate SNPH protein and mRNA levels, which inturn leads to increased invasiveness in glioblastoma cells. Surprisingly, tumors with stabilized HIFα or with deletions resulted in lower expression of SNPH, denoting SNPH’s principal role in metastasis [50]. 

Mitochondria can enormously influence malignant transformation and dictate the tumor plasticity of cancer cells and govern several mechanisms to address tough environmental conditions. Mitochondria are the major source of ROS utilized in OXPHOS. Mitochondrial enzymes, such as pyruvate dehydrogenase (PDH), a-ketoglutarate-dehydrogenase (a-KGDH), acyl-CoA dehydrogenase, and glycerol-3-phosphate dehydrogenase, are involved in ROS generation [51]. The huge difference between normal cells and cancer cells is the controlled levels of mitochondrial ROS (mROS). The levels of mROS are properly regulated in cancer cells in order to play a role in essential cellular processes. On the other hand, cancer cells have functions like oncogene activation, tumor suppressor loss, and hypoxia, which lead to uncontrollable mROS levels that aid in sustaining cancer cell. mROS levels participate in multiple steps of oncogenesis and induce mtDNA mutations. They also influence apoptosis evasion, metabolic reprogramming, and cellular proliferation [52]. mROS is responsible for the activation of several important oncogenic signaling pathways such as the epidermal growth factor receptor (EGFR) signaling pathway [53]. Mitochondria help the epithelial cells in gaining migration speed by providing energy, as it was demonstrated that insufficient energy with deficiencies of mitochondria inhibited cell motility. Similarly, mitEGFR enhances mitochondria fission and cancer cell motility, independent of its phosphorylation status [45]. In order to drive towards a proliferative state and escape mitochondrial permeability transition-mediated cell death, cancer cells intelligently maintain high levels of anti-oxidant proteins to prevent ROS accumulation. On the other hand, the interrelationship between mROS and hypoxia inducible factor-1 (HIF-1) is complex. Hypoxia-mediated mROS leads to HIF-1 activation, which facilitates metastasis because of the metabolic shift from OXPHOS to glycolysis by increasing the expression of glycolyticenzymes. In contrast, HIF-1 decreases mROS production, promotes tumor growth, and facilitates the survival of metastatic cells, denoting the vibrant and functional role of mROS in various cancers [51]. 

Similar to the above, Sirtuin 3 (SIRT3) is involved in several key processes such as the response to oxidative stress and mitochondrial metabolism regulation. SIRT3, a NAD+-dependent mitochondrial deacetylase that promotes efficient oxidative metabolism, is a key regulator of mitochondrial ROS production and detoxification [54]. Literature suggests that SIRT3 has a role in regulating mitochondrial quality control and affects genes involved in homeostasis such as PGC-1α and TFAM. SIRT3 silencing results in making breast and colon cancer cell lines prone to cytotoxic treatment-mediated sensitivity via escalated oxidative stress and altered biogenesis [55]. SIRT3’s role in malignancy was also assessed in a study where silencing of SIRT3 resulted in a reduction of visible clones by 64%, when assessed by a clonogenicity assay. This enumerates the fact that SIRT3 downregulation leads to compromised mitochondrial metabolism and increased sensitivity to oxidative stress [56]. Promotion of metastasis by ROS is tricky. High levels of ROS lead to inhibition of metastasis in melanoma, whereas in other cancers, ROS promotes metastasis [54]. Considering the role of SIRT3 in regulating ROS homeostasis, studies have shown that SIRT3 is essential in extinguishing Src oxidation and Src/Fak signaling to inhibit cell migration and metastasis in breast cancer cells via ROS adjustment [54].

Mitochondrial fission is a process commonly implicated in tumor progression, where dynamin-related protein-1 (Drp1) is bonded to one of its receptors, mitochondrial fission factor (MFF), on the mitochondrial outer membrane. Mitochondrial fission has been widely correlated with cell death and mitochondrial integrity [57]. MFF is overexpressed in numerous cancers. MFF is linked to VDAC1 in the mitochondrial outer membrane, which partially explains its association with cancer progression. However, mechanistically, MFF silencing leads to an upsurge of mitochondrial outer membrane permeability and oxidative stress, which inturn leads to the triggering of mitochondrial-mediated cell death, thereby impeding tumor proliferation and metastasis in mice [57]. A very recent study has determined the role of mitochondrial fission in cancer [58]. This study utilized phosphatidyl serine decarboxylase (PISD), an enzyme that orchestrates mitochondrial fission. It was evidenced that mitochondrial fission inhibits metastasis in triple-negative breast cancer cells. The study also enumerated that the alterations in mitochondrial fission not only inhibited cancer metastasis, cell migration, and cell invasion, but also repressed cancer cell signaling via ERK and Akt [58].

## 5. Mitochondrial Stress Response in Cancer Spread

Mitochondria are responsible for more than just energy production. Recently discovered mechanisms like mitochondrial unfolded protein response (mtUPR) and mitochondrial precursor over accumulation stress (mPOS) are paving new avenues for therapeutics and for understanding diseases better via mitochondria [59]. A genetic study in yeast denoted a novel protective mechanism named mPOS against mitochondrial protein import deficiency. mPOS is a newly-discovered pathway of proteostatic stress-mediated cell death due to mitochondrial dysfunction. mPOS is triggered by mitochondrial damage and the aberrant accumulation of mitochondrial precursors in the cytosol [60]. In parallel to this, mitoCPR was spotted in budding yeast. mitoCPR is a novel cellular response to defective mitochondrial protein import that protects mitochondrial functions [61]. The mitochondrion has an inherently stressful internal environment and it is speculated that dysregulation of stress signaling or an inability to switch on these adaptations during times of mitochondrial stress may underpin mitochondrial dysfunction and amount to pathological states overtime.

The role of mitochondrial chaperones in the cell stress response is quite intriguing. Gamitrinib, for instance, is a mitochondrial targeted HSP90 inhibitor with potential anti-cancer activity. Glioblastoma cells induced with low doses of gamitranib revealed accumulation of unfolded proteins in the mitochondria and a stress response gene characterized by upregulation of chaperones, especially Hsp70. Utilizing this target (mitoUPR) in mitochondria, TRAP-1 or CypD were ablated by genetic or chemical inhibitors. This resulted in the downregulation of NF-kB and related genes. Furthermore, there was an upregulation of pro-apoptotic genes, which aided in mitochondrial-mediated cell death. NF-kB has a wide role in tumor promotion and endorsement in the metastatic environment. Additionally, NF-kB plays a major role in treatment resistance and poor outcomes in cancer. Hence, targeting mitoUPR aids in concomitant loss of NF-kB, which inturn results in exposing the tumors to apoptosis-based therapies [62]. This clearly shows that mitoUPR can be a potential target for cancer therapy. Supplemental to this, new study evidence shows that mitoUPR under the absence of stress, as a part of an adaptive mechanism by cancer cells, results in reduced oxidative stress and is called mitohormesis. mitUPR has an axis with SIRT3, which supports invasion and metastasis. In addition, changes in the mtUPR gene resulted in poor clinical outcomes in patients with breast cancer [63].

## 6. Mitochondrial Ion Channels as a Target in Combating Cancer

The mitochondrial channels, characterized as either outer or inner membrane channels, are widely targeted in cancer therapies. The outer membrane channels include VDAC and the inner membrane channels include mtKATP, mtBKCa, mtIKCa, mtKv1.3, mtTASK-3, and the nonselective permeability transition pore (MPTP) [64]. Mitochondrial outer membrane channels participate in mitochondrial outer membrane permeabilization, while inner membrane channels modulate changes in membrane potential and thereby influence reactive oxygen (ROS) production and efficiency of the respiratory chain. ROS in turn may activate MPTP or the caspase-independent ROS-triggered parthanatos (poly (ADP-ribose) polymerase-1 dependent cell death). In addition, MPTP can also be triggered by Ca2+overload in the mitochondrial matrix or by IMM depolarization and by several other factors (for example oxidative stress) [65]. 

The basis for mitochondrial ion channels being targeted is due to their role in cancer metastasis. A brief description of the role of potassium channels and their role in cancer progression is that channels like IKCa control OXPHOS. Inhibition of the channel has no or only minor effects on cell proliferation in the presence of glucose, but forcing the cells to generate ATP exclusively via oxidative phosphorylation by culturing them in galactose, allowed researchers to understand that inhibition of the channel decreased proliferation. Kv1.3 is another channel that modulates the cell cycle. Mitochondrial calcium fluxes have also been shown to regulate cancer proliferation. Additionally, calcium channels also drive proliferation. The constitutively active Ca2+transfer from the endoplasmic reticulum (ER) to mitochondria plays a crucial role inensuring viability of tumorigenic cells, and defects in this uptake into mitochondria lead to cancer cell death. The crosstalk between potassium and calcium channels isnot completely clear, but a putative K+/H+transporter, LETM1, has been shown on calcium influx/efflux into/from mitochondria, and silencing ofLETM1 promoted AMPK activation, cell cycle arrest, and autophagy [66].

MPTP can be activated indirectly by different drugs eliciting changes in inner membrane potential, causing ROS production, or leading to calcium overload in the matrix. MPTP opening leads to rupture of the mitochondrial outer membrane (MOM), which contributes to cytochrome-c release, a process required for apoptosome formation and subsequent activation of effector caspases.

## 7. Mitochondria as a Therapeutic Target in Cancers

The energy required for cancer cell migration, invasion, and metastasis is supplied by mitochondria. Suppression of the mitochondrial energy function can reduce the frequency of tumor cell metastasis and invasion (Table 1). Targeting dysregulated Drp1-dependent mitochondrial fission could supply a novel scenario for defeating breast cancer metastasis [8]. On the other hand, another novel therapeutic strategy to limit or prevent cancer metastasis is by potentially blocking EMT through targeting specific EMT biomarker genes that are correlated with mitochondria health, signal proteins of the mitochondrial reverse signaling pathway, specific metabolic enzymes, or metabolism-dependent epigenetic reprogramming [33]. As proof, the PI3K/AKT signaling pathway plays a key role in EMT progress and is considered to be a principal signaling pathway in cancer that prompts extensive transcriptional and metabolic reprogramming, specifically in mitochondria. PI3K has been considered a potential target for the prevention and treatment of metastatic tumors. Inhibitors of PI3K have been utilized in tumor treatment to inhibit mitochondrial ATP production and diminish glycolysis [67,68,69]. A recent study highlighted a compound called NSC130362, which belongs to the class of 1,4-naphthoquinones (NQs) and has vibrant pharmacological properties [70]; it has been shown to possess anti-cancer effects, including anti-proliferative and anti-angiogenesis activity [71,72], suppress glycolysis and mitochondrial function [73], and inhibit NF-κB signaling [74]. Natural products on the other hand have also received attention in cancer chemotherapy. Honokiol (HNK) is a potent anti-tumor agent that affects EGFR and mitochondrial function to inhibit the cancer cells’ genesis and metastasis. A study has shown that HNK inhibits mitochondrial respiration, which leads to the induction of apoptosis in lung cancer cells [75]. There are other natural products that have been identified with direct or indirect effects on mitochondrial function in cancers (Table 2 [76]).

## 8. Conclusions

Recent advances in the field of cancer biology have delineated mitochondrial dysfunctions in cancer. Tumors take advantage of the modulated mitochondrial function to escalate invasiveness. Key mechanisms like respiration are not only essential for tumor growth but also for navigating tumor cells into the circulatory system, facilitating metastasis. Mechanisms connecting mitochondrial dynamics to the development of metastasis remain a puzzle. Moreover, the capability of the mitochondria in allowing cancer cells to adapt to stress should be considered. Consequently, mitochondrial biogenesis might answer these questions and unravel the mechanisms useful for therapeutic strategies for cancer treatment.

## Figures and Tables

**Table 1:** Treatment targets of mitochondria to suppress tumor cells metastasis.

Target Treatment	Mechanism	Cancers	References
Phosphatidylinositol-3 kinase (PI3K)	Inhibits mitochondrial transcription and metabolic reprogramming	Lung cancer cell lines	[68]
Adenomatous Polyposis Coli (APC) protein	Reverses mitochondrial trafficking by regulating Wnt signaling	Colorectal cancer	[77]
Drp1, Mnf1	Extends mitochondrial fission	Breast cancer	[8]
pSer9-GSK-3β;	Suppresses mitochondrial respiratory chain complexes	Breast cancer	[78]
Mito-TAM (derivative of Tamoxifen)	Disrupts mitochondrial respiratory chain complexes and OXPHOS	Breast cancer	[79]
PGC-1α	Impairs mitochondrial biogenesis and OXPHOS	shPGC-1α cells	[27]

**Table 2:** Natural compounds affecting mitochondrial function and acting as cancer therapeutics [76].

Compound	Source	Mode of Action	Cancers
Honokiol	*Magnolia grandifloris*	Induces mitochondrial apoptosis	Lung cancer, Breast cancer, Leukemia
Curcumin	Turmeric	Inducesapoptosis via multiple mechanisms	Skin cancer, Cervical cancer, NSCLC
Pancratistatin	Spider lily *Pancratiumlittorale*	Induces ROS stress, loss of mitochondrial potential, apoptosis	Breast cancer, Colon cancer, Lymphoma
OSW-1	*Ornithogalumsaudersiae*	Damages mitochondrial membranes, Ca2+ dependent apoptosis	Leukemia, Malignant brain tumor, Pancreatic cancer
Epigallocatechin-3-gallate (EGCG)	Green tea	Accumulates in mitochondria, inducing apoptosis	Breast cancer, Colon cancer, Pancreatic cancer, Melanoma
Vitamin K3	Synthetic vitamin K precursor	Inhibits mitochondrial pol γ, causing ROS stress	Leukemia and various solid tumors

## Abbreviations

OXPHOS	Oxidative phosphorylation
SNP	Single nucleotide polymorphisms
EMT	Epithelial mesenchymal transition
Drp1	Dynamin-related protein
Mfn	Mitofusion
ROS	Reactive oxygen species
EGFR	Epidermal growth factor receptor
NSCLC	Non-small cell lung cancer
AML	Acute myelogenous leukemia
STUB1	STIP1 homology and U-Box containing protein 1
USP7	Ubiquitin-specific-processing protease 7
PDH	Pyruvate dehydrogenase
a-KGDH	a-ketoglutarate-dehydrogenase
Drp1	Dynamin-related protein-1
VDAC	Voltage dependent anion channels
MFF	Mitochondrial fission factor
PISD	Phosphatidyl serine decarboxylase
SIRT	Sirtuin
PI3K	Phosphatidylinositol-3 kinase
HNK	Honokiol
MOM	Mitochondrial outer membrane
